# Molecular Dynamics Studies of the Mechanical Behaviors and Thermal Conductivity of Polyisoprene with Different Degrees of Polymerization

**DOI:** 10.3390/polym14224950

**Published:** 2022-11-16

**Authors:** Zhiyuan Chen, Qunzhang Tu, Zhonghang Fang, Xinmin Shen, Qin Yin, Xiangpo Zhang, Ming Pan

**Affiliations:** Field Engineering College, Army Engineering University of PLA, Nanjing 210007, China

**Keywords:** natural rubber, polyisoprene, molecular dynamics simulation, polymerization, uniaxial tension, thermal conductivity

## Abstract

Polyisoprene, with a high degree of polymerization, is the main component of natural rubber. In the industrial production process, it is necessary to adjust the length of the macromolecule of polyisoprene to improve its plasticity. It is thus of vital importance to explore the effect of the degree of polymerization of polyisoprene on its properties, e.g., mechanical property and thermal property. Molecular dynamics simulations link microstructure to macroscopic properties. In this paper, Moltemplate was used to establish polyisoprene models with different degrees of polymerization, and the mechanical properties of polyisoprene under uniaxial tension were analyzed under an OPLS all-atom force field. The results showed that the strength and elastic modulus of the material increased with the increase in the degree of polymerization of the molecular chain. In the process of tensile loading, the non-bonded potential energy played a dominant role in the change of the total system potential energy. Then, the thermal conductivity of polyisoprene with different degrees of polymerization was calculated by the non-equilibrium molecular dynamics method (NEMD). The thermal conductivity of PI was predicted to converge to 0.179 W/(m·K). The mechanism of thermal conductivity of the polymer containing branched chains was also discussed and analyzed. The research content of this paper aims to provide theoretical support for improving the mechanical and thermal properties of natural rubber base materials.

## 1. Introduction

Natural rubber is a kind of natural polymer material, with a high degree of polymerization, which is widely used in the production of tires, conveyor belts, sealing rings, and other rubber products [1]. The main component of natural rubber is cis-1,4-polyisoprene whose relative molecular weight is approximately 10^5^~10^6^ g/mol [2,3,4]. Relatively high molecular weight has an adverse effect on the processing of natural rubber. In order to reduce the molecular weight of natural rubber and improve its processing performance, natural rubber needs to be plasticized [5]. However, excessive plasticizing can reduce the strength, elasticity, wear resistance, and thermodynamic properties of its products. Therefore, exploring the influence of the relative molecular weight of polyisoprene on its mechanical and thermal properties has important significance for guiding the production and preparation of high-performance natural rubber products [6,7,8].

Molecular dynamics simulation (MD) combines classical mechanics with statistical mechanics to calculate and analyze the mechanical properties of materials and establishes the relationship between microstructure and macroscopic properties [9,10]. This means that researchers can design new materials and analyze their performance through molecular dynamics simulations without conducting trial-and-error-based experiments [11,12].

In order to study the mechanical properties of polymers by the MD method, the correlation was established between the molecular model and the continuum model [13,14]. Furthermore, Uddin et al. [15] developed a natural rubber constitutive model based on the rubber network theory and MD simulation. Kitamura et al. [16] used MD simulation to study the mechanical behavior of polypropylene rubber under uniaxial tensile, uniaxial compressive, biaxial tensile, and biaxial compressive loading conditions. Payal et al. [17] studied the tensile deformation of polybutadiene rubber based on all-atom molecular dynamics simulation and analyzed the material destruction process during the tensile process.

On the other hand, there are also many studies that calculate the thermal conductivity of polymers by MD simulation [11,18,19]. Xiong et al. [20] studied the thermal conductivity of cross-linked polyethylene with the effect of crosslink degree, chain length, and strain. The result mentioned was that thermal conductivity increased as crosslink degree and chain length increased. Zhao et al. [21] found that the thermal conductivity of polymers increases with increasing the molecular weight. Terao et al. [22] investigated the effect of simulation time step on the thermal conductivity of the polymer system and found that, no matter if the time step was 1.0 fs or 0.5 fs, the calculated thermal conductivity had no significant difference.

The description of thermal conductivity is more complicated for polymers containing branched chains. Because of the disordered arrangement of the molecular chain segments, the crystallization ability of the branched polymer chain is generally weak. Thus, there are more amorphous components and an incomplete crystal structure appears in its molecular structure [23]. For example, Pal et al. [24] found that the heat transfer of polymer materials requires the homogeneity of atomic mass through the study of the thermal conductivity of amorphous blends. Similarly, Luo et al. [25] found that the heat transfer of polymer materials generally requires high regularity and symmetry of space atoms by studying the thermal conductivity of PDMS single-molecule chains. Therefore, the thermal conduction mechanism of the polymers containing branched chains still needs to be explored. Meanwhile, the factors affecting the thermal conductivity of the polymer need to be studied.

In this paper, Moltemplate software [26] was used to establish polyisoprene models with different degrees of polymerization. The mechanical properties of polyisoprene under uniaxial tension were analyzed under the action of an OPLS (optimized potentials for liquid simulations) all-atom force field [27,28,29], and the influence of polymerization degree on its mechanical properties was explored. To explore the thermal conductivity of the polymers containing branched chains, polyisoprene with different degrees of polymerization (*p* = 50, 100, 150, and 200, Mw = 3304, 6604, 9904, and 13,204 g/mol, respectively) was calculated by the non-equilibrium molecular dynamics method (NEMD) and compared with that of polybutadiene. Then, the mechanism of thermal conductivity of the polymer containing branched chains was discussed and analyzed. The research content of this paper aims to provide theoretical support for improving the mechanical and thermal properties of natural rubber base materials.

## 2. Materials and Methods

### 2.1. Construction of the Simulation Model

**Figure 1:**
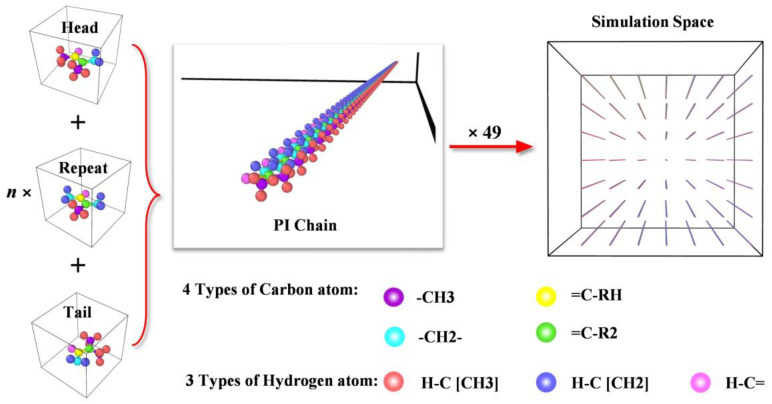
Model process of PI molecular model.

The main component of natural rubber is cis-1, 4-polyisoprene. In this article, a polyisoprene (PI) molecular model was established by Moltemplate. As depicted in Figure 1, the molecular model of the head group, repeat group, and tail group were established at first. Then, a head group, a tail group, and several repeat groups were connected to form a PI long chain with a certain degree of polymerization. Finally, a total of 49 PI long chains were arranged equally spaced in an extremely large cube space.

**Figure 2:**
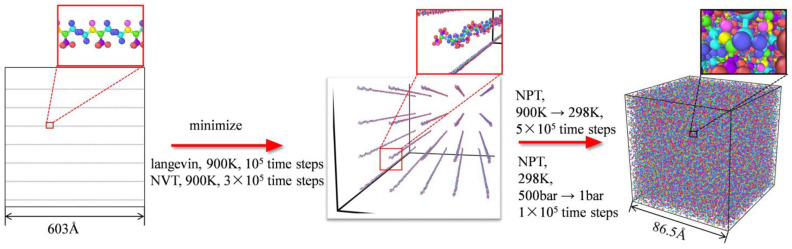
Molecular model of polyisoprene after equilibrating.

The molecular model established above was absolutely unrealistic; not only the size of the simulation box but also the spacing between the molecules were unrealistically large. Therefore, a three-step equilibration process was required before tension simulation and thermal conductivity calculation. The process (*p* = 100, for example) is vividly illustrated in Figure 2. Firstly, an energy minimization of the system by the LAMMPS command “minimize” was performed. Secondly, the molecules at high temperature and constant volume, which would otherwise be pointing in the same direction, were reoriented. During this process, the simulation ran for 1 × 10^5^ time steps using Langevin dynamics at 900 K followed by relaxation for 3 × 10^5^ time steps using NVT dynamics at 900 K under a Nose–Hoover’s thermostat. Thirdly, the simulation ran for 5 × 10^5^ time step to cool the system from 900 K to 298 K, and successively ran for 1 × 10^5^ time steps to decrease the pressure to one bar. In the third step, the simulation ran under NPT conditions to allow the simulation box to contract to natural size at ambient temperature. After finishing the above process, a much more realistic molecular model of polyisoprene was fabricated, which was the initial model for tension simulation and thermal conductivity calculation simulation.

It should be noted that the above equilibrium process used an OPLS all-atom force field [27,28,29] which will be further introduced in Section 2.2.

### 2.2. Interatomic Potential

The OPLS all-atom force field can accurately describe the interaction between atoms in the macromolecule. It is worth noting that OPLS takes all of the interactions between atoms, and the functional form of OPLS has no cross-terms between the different types of terms in the force field. This leads to a more accurate simulation result [30]. Second, much of the literatures regarding molecular dynamics simulations of rubber have used the OPLS potential, e.g., reference [30,31,32]. Thus, this paper can compare the results with those previously obtained. According to this force field, the total potential energy of the system can be expressed as

(1) $$E=E_{bond}(r)+E_{angle}(\theta )+E_{dihedral}(\phi )+E_{non-bonding}$$

As shown in the above equation, the total force field energy had four components: bond stretching, angle bending, dihedral angle torsion, and non-bonded interactions. Among them, the first three terms were collectively called bonded terms, whose functional form were provided by:

(2) $$E_{bond}(r)=K_{b}{\left(r-r_{0}\right)}^{2}$$

(3) $$E_{angle}(\theta )=K_{\theta }{\left(\theta -\theta_{0}\right)}^{2}$$

(4) $$E_{dihedral}(\phi )=\frac{1}{2}K_{1}[1+\cos(\varphi )]+\frac{1}{2}K_{2}[1-\cos(2\varphi )]+\frac{1}{2}K_{3}[1+\cos(3\varphi )]+\frac{1}{2}K_{4}[1-\cos(4\varphi )]$$

where *K_b_*, *K_θ_*, *K*_1_, *K*_2_, *K*_3,_ and *K*_4_ are the stiffness constants, *r*_0_ is the equilibrium bond distance, and *θ*_0_ is the equilibrium value of the angle.

The non-bonded interactions were given as

(5) $$E_{non-bonding}(r)=4\varepsilon [{\left(\frac{\sigma }{r}\right)}^{12}-{\left(\frac{\sigma }{r}\right)}^{6}],\;r<r_{c}$$

where *r* represents the distance between two atoms, *r_c_* is the cutoff distance, *ε* is the distance at zero potential energy, and *σ* represents the energy well depth. The above parameters had already been defined in the OPLS all-atom force field [33].

### 2.3. Uniaxial Tensile Simulation

The equilibrated structure accessed from Section 2.1 was then inserted into the molecular dynamics code to perform a uniaxial tensile deformation. By expanding the distance between two opposite sides of the simulation box at a certain rate, the deformation simulation was operated in LAMMPS. The expanding rate was the uniaxial tensile strain rate, which is set as 1 × 10^10^/s in this article. During the deforming process, not only the stress but also the bond length, bond angles, dihedral angle, and non-bonding interactions were recorded in order to analyze the deformation mechanisms under uniaxial stretch. It should be noted that an NPT ensemble was used to control the temperature of the simulated system at 298 K.

### 2.4. Thermal Conductivity Calculation

**Figure 3:**
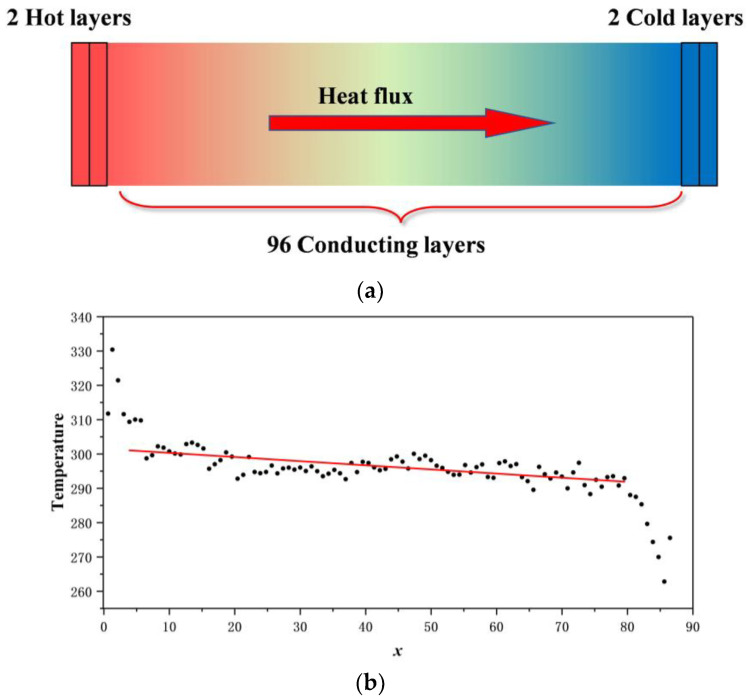
NEMD method for thermal conductivity: (**a**) Schematic diagram of simulation; (**b**) the temperature distribution in the simulation domain and line fitting.

In this article, we calculated the thermal conductivity of PI using the NEMD method [22]. Firstly, fixed boundary conditions were set in the *X* direction, while periodic boundary conditions were used in the *Y* and *Z* directions. Then, the box was equally divided into 100 layers along the *X* direction. As depicted in Figure 3, the first two layers on the left were set as the hot source, where heat was provided 1 eV per 10 time steps. Similarly, the last two layers on the right were set as the cold source, where heat was extracted 1 eV per 10 time steps. By applying heat/cold flow perturbation at two opposite sides of the simulation box, a temperature distribution formed inside the simulation box.

After a certain time, 5 × 10^5^ time steps in this article, the simulation system reached a steady state. As illustrated in Figure 3b, the temperature distribution in the structure could be counted as

(6) $$\nabla T=\frac{\partial T}{\partial x}$$

where ∇T is the temperature gradient, *T* represents the temperature, and *x* represents the displacement of heat transfer direction.

Finally, the thermal conductivity could be calculated by Fourier law, as

(7) $$\lambda =-\frac{J}{A\times \nabla T}$$

where *λ* represents the thermal conductivity, *A* represents the heat transfer area, and *J* is the heat flux, which is provided as

(8) $$J=\frac{\partial E}{\partial t}$$

where *E* represents the input energy and *t* represents the simulation time.

## 3. Results and Analysis of Tensile Simulation

### 3.1. Stress–Strain Behavior

**Figure 4:**
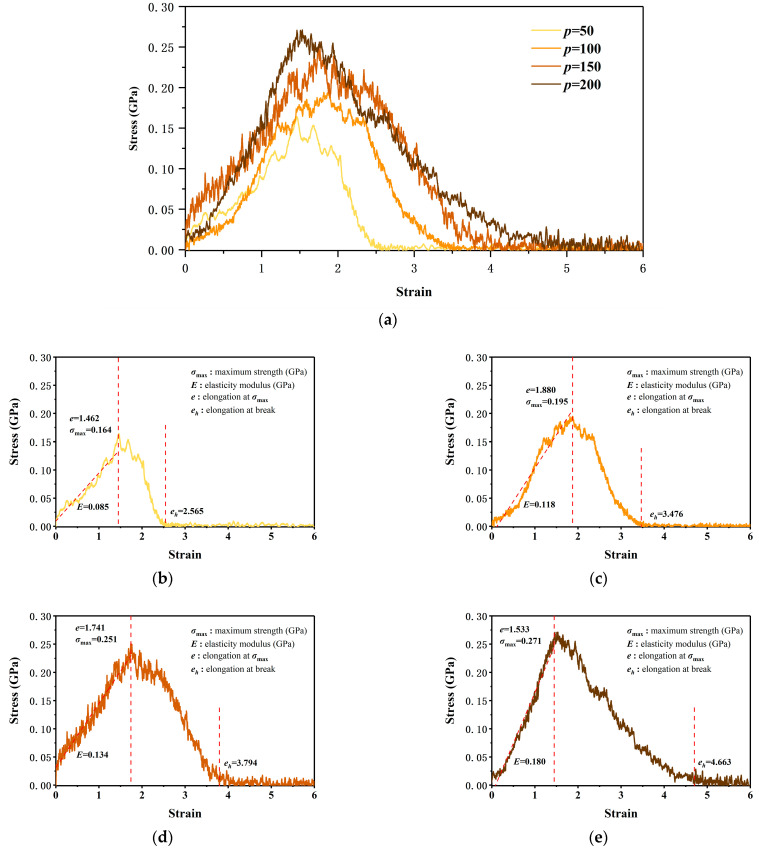
Stress–strain curve of PI: (**a**) overall; (**b**) *p* = 50; (**c**) *p* = 100; (**d**) *p* = 150; (**e**) *p* = 200.

The stress–strain curves of PI with different polymerization are illustrated in Figure 4. As depicted in Figure 4a, the material behaved linearly elastic at the initial time. Then, stress dropped down after reaching the yield point. Finally, the material entered the failure stage and stress decreased until fracture occurs.

From Figure 4a, it can be seen that the stress peak of the material increased with the increase in polymerization degree *p*. To meticulously analyze the mechanical properties of PI during the tension process, the stress–strain curves were plotted separately for each case: *p* = 50, 100, 150, and 200. Maximum strength (σ_max_), elasticity modulus (*E*), elongation at σ_max_ (*e*), and elongation at break (*e_h_*) were annotated on each figure. Detailed data is exhibited in Table 1.

**Figure 5:**
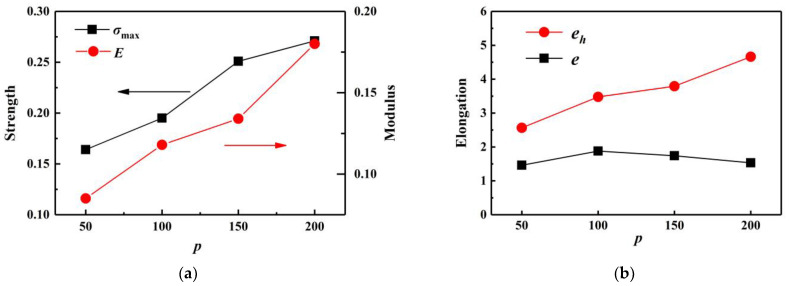
Mechanical property parameters as a function of *p*: (**a**) maximum strength and elasticity modulus; (**b**) elongation at σ_max_ and at break.

To visualize the change trend of the above parameters, Figure 5 depicts the maximum strength, elasticity modulus, elongation at σ_max_, and elongation at break as a function of *p*, respectively. It can be concluded from Table 1 and Figure 5 that σ_max_, *E*, and *e_h_* increased as *p* increased. As *p* escalated, σ_max_ increased from 0.164 GPa to 0.271 GPa, the value of *E* steadily raised from 0.085 GPa to 0.180 GPa, and *e_h_* increased from 256.5% to 466.3%. However, the value of *e* was not sensitive to *p*. In the above four cases, the value of *e* was always approximately 160%. In other words, under uniaxial tensile action, the maximum stress of PI usually appeared at 160% strain.

As the degree of polymerization decreased, the plasticization of PI increased, while the maximum strength, modulus, and elongation at break all decreased. Thus, polyisoprene should be processed according to its target product to equilibrate its properties and plasticization.

### 3.2. Internal Energy Analysis

**Figure 6:**
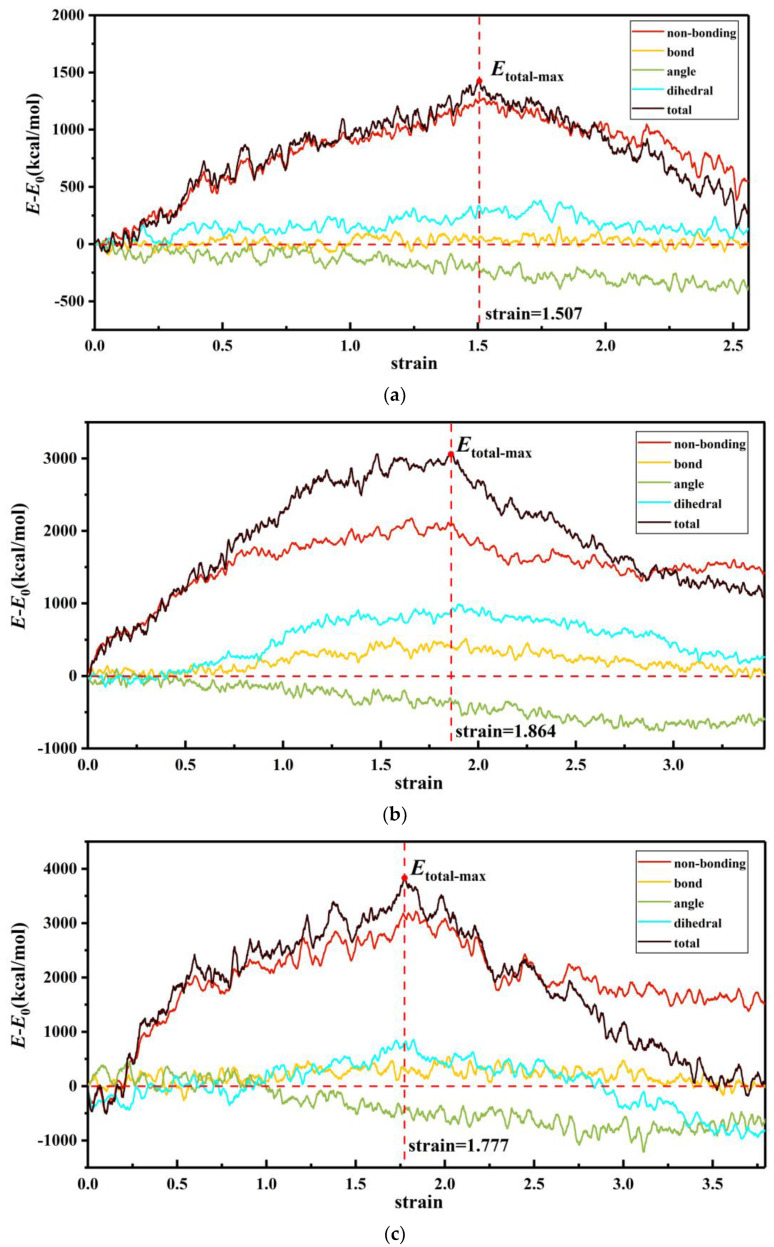

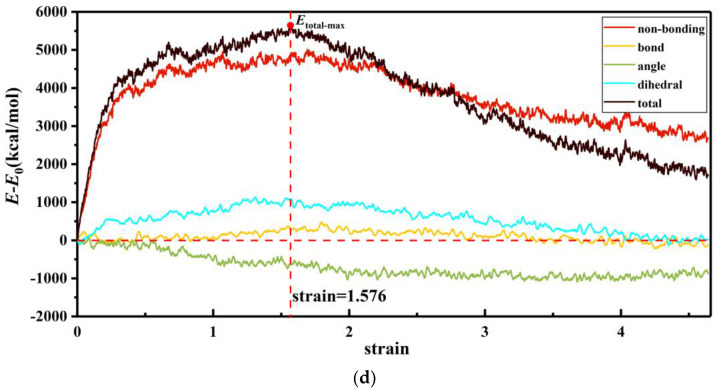
Energy decomposition for the PI system with different degrees of polymerization: (**a**) *p* = 50, (**b**) *p* = 100, (**c**) *p* = 150, (**d**) *p* = 200.

Figure 6 shows the variation of non-bond potential energy increment, bond-stretching energy increment, angle-bending potential energy increment, dihedral angle torsion potential energy increment, and total-system potential energy increment with strain in the process of uniaxial tension of the PI system.

As depicted in Figure 6, the energy evolution of the PI system with different degrees of polymerization followed a similar trend.

Table 2 lists the proportion of each energy component in the total system energy when the system energy was maximum (the position marked by the vertical red dashed line). Obviously, the increase of the total potential energy of the system was mainly attributed to the non-bonded potential energy during the uniaxial tensile process of the PI system, and the bond-stretching energy, angle-bending potential energy, and dihedral angle torsion potential energy contributed less than that of the non-bonded potential energy. Among them, the non-bonded potential energy of the system increased in a low strain range, which corresponded to the elastic deformation range, and decreased after reaching the maximum strength point. The dihedral angle torsion potential energy had a similar variation trend with the non-bond potential energy, but the increment of the dihedral angle torsion potential energy was lower than that of the non-bond potential energy. During the whole deforming process, the angle-bending potential energy gradually dwindled, while the bond-stretching energy changed slightly.

In addition, the peak of non-bonding energy was positively correlated with the degree of polymerization, which suggested stronger inter-chain interactions. This explained that the strength and modulus increased as the degree of polymerization increased in terms of molecular energy. These results validated that the MD method was reliable to study the mechanical properties of PI.

## 4. Results and Analysis of Thermal Conductivity

**Figure 7:**
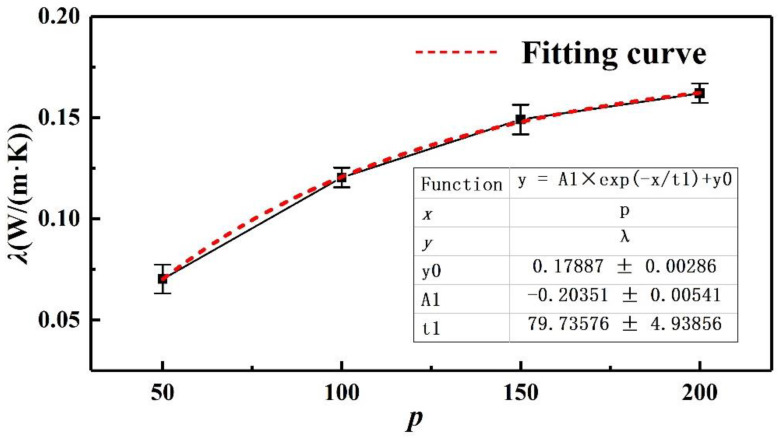
The change of PI thermal conductivity with the degree of polymerization.

It was reported that the thermal conductivity of polymers had a significant relationship with the length of the molecular model [25]. In our study of polyisoprene, we found a similar result that the thermal conductivity of polyisoprene had scale effect. It is illustrated in Figure 7 that the thermal conductivity of polyisoprene increased with the increase in *p*, and the increase in amplitude gradually slowed down. 

When the degree of polymerization *p* < 150, the increase rate of thermal conductivity is relatively large, and the thermal conductivity increases approximately linearly. According to Liu et al. [34], the heat transfer mode belonged to ballistic phonon heat transfer when *p* was small. In this mode, the phonon was more susceptible to the interference of scattering in the cold and hot areas at the boundary and transfers less heat. Therefore, the thermal conductivity was lower. When *p* increased with the system size increasing, the system of heat transfer changed from the ballistic phonon heat transfer to diffuse phonon heat transfer, also called the ballistic diffusion phonon heat transfer. In the process of heat transfer, the system receives less scattering interference from the boundary, and the heat transfer increases, so the thermal conductivity increases. However, the diffusion phonon heat transfer was realized by the collision of phonons and phonons. The increase of the phonon collision probably decreased the phonon mean free path. Therefore, the increasing trend of thermal conductivity gradually slows down. Based on the above variation of thermal conductivity, the exponential function was selected to fit the data, and a good fitting result was obtained, as shown in Figure 7. The thermal conductivity of PI was predicted to converge to 0.179 W/(m·K), which was approximately the same as the experimental results in reference [35] (0.18 W/(m·K)) and reference [36] (0.168 W/(m·K)). This indicated that the prediction of PI thermal conductivity by the MD method was reliable.

According to quantum mechanics [37], one of the factors affecting the size of the phonon mean free path of the system was the scattering of phonons by the defects in the solid, such as the inhomogeneity of the crystal, the boundary of the polycrystal, and the impurities. According to phonon heat transfer theory and ideal gas dynamics theory, the thermal conductivity of polymer materials has the following relationship with heat capacity *C*, sound velocity *v,* and phonon mean free path *L*:

(9) $$\lambda =\beta c\bar{v}L$$

where *β* represents the dimension characteristics of materials. *β* = 1/3 for isotropic three-dimensional crystal materials, *β* = 1/2 for isotropic two-dimensional crystal materials, and *β* = 1 for one-dimensional materials. Compared with polybutadiene, the branched chains in polyisoprene destroy the symmetry of the polymer chain, resulting in the lattice inhomogeneity of the system. Therefore, phonons are prone to collision, which restricts the mean free path L of phonons. Therefore, the thermal conductivity of polyisoprene was lower than that of straight chain polymers, such as polyethylene and polybutadiene [32].

## 5. Conclusions

In this study, all-atom molecular models of polyisoprene with different degrees of polymerization were established. Based on the OPLS all-atom force field and NEMD method, the mechanical and thermal properties of the material were tested by molecular dynamics simulation. During uniaxial tensile deformation, the strength and elastic modulus of polyisoprene increased with the increase in the molecular chain polymerization degree. Through the analysis of the system energy in the deformation process, it could be found that the non-bonded potential energy played a dominant role in the change of the total potential energy of the system. As the degree of polymerization increased, the increase of PI thermal conductivity became inapparent, which could be well fitted by exponential function. As a result, the thermal conductivity of PI was predicted to converge to 0.179 W/(m·K). Compared with polyethylene and polybutadiene, polyisoprene had low thermal conductivity and a more obvious size effect due to the branch in the main chain. The results of this paper could provide theoretical support for improving the mechanical and thermal properties of natural rubber base materials.

## Figures and Tables

**Table 1:** Mechanical property parameters of PI with different *p*.

Parameters	*p* = 50 *(M_w_* = 3304)	*p* = 100 *(M_w_* = 6604)	*p* = 150 *(M_w_* = 9904)	*p* = 200 *(M_w_* = 13,204)
Maximum strength (GPa)	0.164	0.195	0.251	0.271
Elasticity modulus (GPa)	0.085	0.118	0.134	0.180
Elongation at σ_max_	146.2%	188.0%	174.1%	153.3%
Elongation at break	256.5%	347.6%	379.4%	466.3%

**Table 2:** The proportion of each energy component at the maximum of total system energy.

Parameters	*p* = 50 *(M_w_* = 3304)	*p* = 100 *(M_w_* = 6604)	*p* = 150 *(M_w_* = 9904)	*p* = 200 *(M_w_* = 13,204)
strain	1.507	1.864	1.777	1.576
bond	2.57%	13.29%	8.26%	4.95%
angle	−11.93%	−11.58%	−10.39%	−11.29%
dihedral	22.25%	30.09%	21.32%	18.82%
non-bonding	87.11%	68.21%	80.81%	87.52%

## Data Availability

The data that support the findings of this study are available from the corresponding author upon reasonable request.
